# Effects of the Training Dataset Characteristics on the Performance of Nine Species Distribution Models: Application to *Diabrotica virgifera virgifera*

**DOI:** 10.1371/journal.pone.0020957

**Published:** 2011-06-20

**Authors:** Maxime Dupin, Philippe Reynaud, Vojtěch Jarošík, Richard Baker, Sarah Brunel, Dominic Eyre, Jan Pergl, David Makowski

**Affiliations:** 1 INRA, UR Zoologie Forestière, Ardon, Orléans, France; 2 CIRAD, UMR Peuplement Végétaux et Bio agresseurs en Milieu Tropical, Montpellier, France; 3 Anses, Laboratoire de la Santé des Végétaux, Station d'Angers, Angers, France; 4 Department of Ecology, Faculty of Science, Charles University, Prague, Czech Republic; 5 Food and Environment Research Agency, Sand Hutton, York, United Kingdom; 6 EPPO/OEPP, Paris, France; 7 Institute of Botany ASCR, Průhonice, Czech Republic; 8 Institute of Ecology and Evolution, University of Bern, Bern, Switzerland; 9 INRA, UMR 211 INRA AgroParisTech, Thiverval-Grignon, France; National Institute of Water & Atmospheric Research, New Zealand

## Abstract

Many distribution models developed to predict the presence/absence of invasive alien species need to be fitted to a training dataset before practical use. The training dataset is characterized by the number of recorded presences/absences and by their geographical locations. The aim of this paper is to study the effect of the training dataset characteristics on model performance and to compare the relative importance of three factors influencing model predictive capability; size of training dataset, stage of the biological invasion, and choice of input variables. Nine models were assessed for their ability to predict the distribution of the western corn rootworm, *Diabrotica virgifera virgifera*, a major pest of corn in North America that has recently invaded Europe. Twenty-six training datasets of various sizes (from 10 to 428 presence records) corresponding to two different stages of invasion (1955 and 1980) and three sets of input bioclimatic variables (19 variables, six variables selected using information on insect biology, and three linear combinations of 19 variables derived from Principal Component Analysis) were considered. The models were fitted to each training dataset in turn and their performance was assessed using independent data from North America and Europe. The models were ranked according to the area under the Receiver Operating Characteristic curve and the likelihood ratio. Model performance was highly sensitive to the geographical area used for calibration; most of the models performed poorly when fitted to a restricted area corresponding to an early stage of the invasion. Our results also showed that Principal Component Analysis was useful in reducing the number of model input variables for the models that performed poorly with 19 input variables. DOMAIN, Environmental Distance, MAXENT, and Envelope Score were the most accurate models but all the models tested in this study led to a substantial rate of mis-classification.

## Introduction

Since the 1950’s, biological invasions have increased due to an intensification of global trade [1], [2], [3]. The establishment and spread of invasive alien species has led to important economic and environmental damage [4], [5], [6]. The risks of damage are likely to rise with increasing global trade and in an era with a rapidly changing climate. In Europe alone, 11000 alien species have invaded, and 30% of them led to economic damage or have caused harm to biological diversity [7].

Species distribution models (SDMs) or ecological niche models provide a basis for predicting the distribution potential of invasive species in regions other than their native ranges [8]. Although these models do not answer the associated questions of opportunities for entry, evolutionary bottlenecks, potential for spread, and impact, SDMs can be used to anticipate the geographical course of species’ invasions [8]. Peterson and Vieglais [9] and Peterson [8] explored the ability of species distribution models (SDMs) to characterize the climatic conditions that are suitable for invasive alien species and to identify new areas where these species could establish. They concluded that these models provide proactive, predictive, and quantitative tools for pest risk analysis (PRA). SDMs can also be coupled to climate change models to predict how the geographic ranges of species will shift as anthropogenic climate change proceeds [10], [11].

Some of these models, like CLIMEX [12] or the NAPPFAST phenology and generic infection model [13], can incorporate climatic tolerance data from laboratory studies or may infer parameter values from the relationship between the distribution and the climate. The accuracy of the predictions from these models is influenced by the aptitude, knowledge, training and time input of each modeller as well as the data available. Other SDMs use a correlative approach based on a wide variety of statistical methods or machine-learning techniques to assess climatic suitability. These models implement classification rules developed from a training dataset including a set of georeferenced presence locality records and values of climatic variables for each site usually obtained from interpolated climatic data sets. A great diversity of SDMs is available, and it is important to compare the performance of these models to help risk assessors to choose the one that is most appropriate.

Several studies have been carried out to assess and compare SDM performance [14], [15], [16]. The authors showed that no model was systematically better than the others and that no single model was optimal for all applications and species. Therefore, an increased insight into the performance of models should help to provide guidance on which are more appropriate in different situations. SDM performance is likely to depend on the size of the training dataset [16], [17], [18], [19], [20], [21], [22] and on the geographical spread of the presence locations [23], [24], [26], [27], [28], [25] used for model calibration. These two factors depend themselves on the stage of the biological invasion. The size of the training dataset and the spread of the presence locations are more likely to be restricted at an early stage of a biological invasion than at a late stage. The choice of the input variables is another important consideration to take into account when assessing the performance of modelling methods as the selected set of input variables and their correlations can influence model performance [29], [30], [33], [34], [35]. To our knowledge, the combined effects of all these factors (i.e., size of training dataset, stage of the biological invasion, and choice of input variables) have not been analysed and their relative importance have not been compared.

Some SDMs can be calibrated using species presence data only, whereas others require either true absence or ‘pseudo-absence’ records in addition to presence records. The type of absence data can influence model prediction accuracy [31], [32]. Pseudo absence locations are points or pixels randomly selected from an area around presence records from which the species being modelled is not known to occur as opposed to known not to occur as a result of a survey. Vaclavik and Meentemeyer [31] found that models performed better when calibrated using true absence data when dispersal constraints were taken into consideration. However, true absence data are not frequently available for invasive species and, in practice, SDMs are calibrated using pseudo-absence data [31]. True-absence data have been recognized to be a critical ingredient not only for model calibration but also for model assessment. When pseudo-absence data are used instead of real absence data for model assessment, the levels of accuracy of the tested models can be over-estimated [31]. Although models were assessed using pseudo-absence data in several comparative studies in the past ([21], [28]), it is more appropriate to use reliable absence data to assess the accuracy of SDMs [31].

The aim of this study was to investigate the effects of the size of the training dataset, of the stage of the biological invasion, and of the number of input bioclimatic variables on the performance of nine distribution models. Model comparisons were performed using *Diabrotica virgifera virgifera* presence and absence data from North America and Europe. Numerous surveys have been carried out for this species using pheromone traps and a very large number of reliable presence and absence records are thus available to assess SDM predictions for this invasive insect. Several training data sets were defined at two different stages of *D. virgifera virgifera* invasion (invaded areas recorded in 1955 and 1980). The nine models studied were first fitted to each training dataset in turn using three different sets of input variables, and model performance was then assessed using independent data. Models were calibrated using presence and pseudo-absence data as usually done in practice for invasive species, but model assessment was performed using recorded presence and absence data as recommended by [31]. Results were used to rank the models according to the size of the training dataset, the stage of the biological invasion, and the number of bioclimatic input variables.

## Materials and Methods

### Presence and absence data of the Western Corn Rootworm in North America and Europe

The western corn rootworm (WCR), *Diabrotica virgifera virgifera* LeConte (Coleoptera: Chrysomelidae), is a major pest of cultivated corn, *Zea mays* L. Most of the damage to this crop is caused by larvae feeding on the roots of maize [36]. This pest species probably originated in Central America but the current southernmost limit of its distribution is in northern Mexico. In the 1950s and 1960s, WCR rapidly expanded its range from the southwestern region of the US Corn Belt, reaching the east coast of North America during the 1980s [37]. It was recently introduced into Europe, where it was first observed near Belgrade, Serbia in 1992 [38].

**Figure 1:**
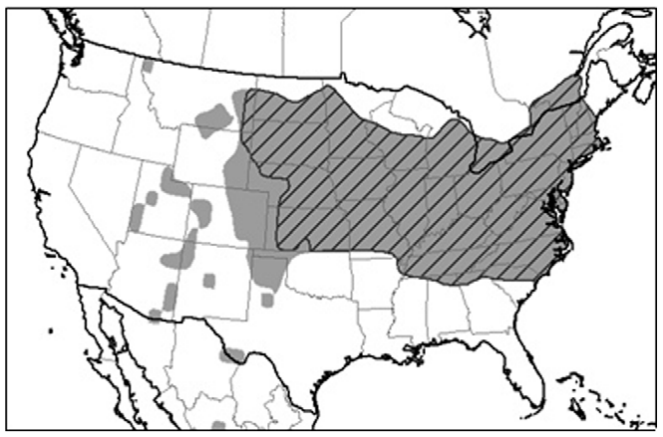
Western corn rootworm distribution in North America. The hatched area represents non-irrigated maize area.

For North America (Figure 1), WCR locations were collected from maps of the Entomology Department at Purdue University (http://extension.entm.purdue.edu/wcr/), drawn by C. Richard Edwards. The 2008 map was used, based on NAPIS and state data. This map was georeferenced using the digital map of US Census Bureau (http://www.census.gov/tiger/tms/gazetteer/county2k.txt). A few additional locations were added based on data reported in the literature. Presence and absence data were derived from WCR occurrence reports from individual counties. Counties with more than 50 percent of the corn acreage with irrigation according to USDA National Agriculture Statistics Service (http://www.agcensus.usda.gov/Publications/2007/index.asp) were considered as irrigated (Figure 1). Only non-irrigated counties were considered as irrigation was not taken into account by the models considered in this paper. Model predictions were meant to be of where the insect would survive in the absence of irrigation.

**Figure 2:**
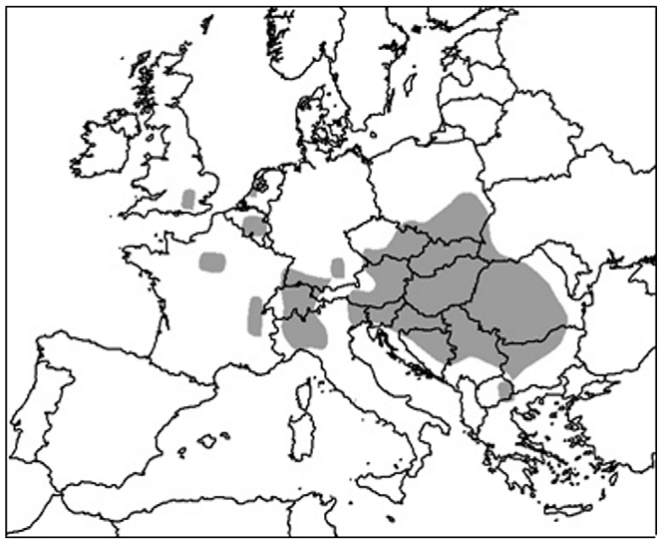
Western corn rootworm distribution in Europe.

For Europe (Figure 2), locations were supplied by National Plant Protection Organizations, by the European and Mediterranean Plant Protection Organization (http://www.eppo.org/) in 2010 and literature was checked for additional locations. Only presence data were considered in Europe as the range of the pest is still expanding and hence absences from this region may not be an indication that the climate is unsuitable.

The total number of presence data points was 1198 (240 in Europe) and the total number of absence data points was 977.

### Climatic data and model input variables

Three sets of input variables were defined; a set including 19 bioclimatic variables, a set including six variables selected among the 19 bioclimatic variables using information on insect biology, and a set of three linear combinations of 19 bioclimatic variables derived from Principal Component Analysis (PCA).

#### Set of 19 bioclimatic variables

Monthly mean temperatures and monthly precipitation sums from the CRU TS 2.1 dataset were used [39], provided by the Climatic Research Unit (CRU, University of East Anglia, Norwich, GB). This dataset includes climatic data for the 1961–1990 period at a 0.5° by 0.5° spatial resolution. A set of 19 bioclimatic variables was defined using DIVA-GIS v5.2 by combining monthly mean temperatures, monthly precipitation sums, or both monthly mean temperatures and precipitation sums. These variables are defined in Table 1.

**Table 1:** Bioclimatic variables computed from monthly mean temperatures (T), from monthly precipitation sums (P), or from both (T + P).

Code	Bioclimatic variables	Initial climatic variable
**Bio01**	**Annual Mean Temperature**	**T**
Bio02	Mean Diurnal Range [Mean of monthly (max temp - min temp)]	T
Bio03	Isothermality [(Bio02/Bio07)*100]	T
Bio04	Temperature Seasonality [standard deviation *100]	T
Bio05	Max Temperature of Warmest Month	T
**Bio06**	**Min Temperature of Coldest Month**	**T**
**Bio07**	**Temperature Annual Range [Bio05–Bio06]**	**T**
Bio08	Mean Temperature of Wettest Quarter	T + P
Bio09	Mean Temperature of Driest Quarter	T + P
Bio10	Mean Temperature of Warmest Quarter	T
**Bio11**	**Mean Temperature of Coldest Quarter**	**T**
Bio12	Annual Precipitation	P
**Bio13**	**Precipitation of Wettest Month**	**P**
Bio14	Precipitation of Driest Month	P
Bio15	Precipitation Seasonality [Coefficient of Variation]	P
Bio16	Precipitation of Wettest Quarter	P
**Bio17**	**Precipitation of Driest Quarter**	**P**
Bio18	Precipitation of Warmest Quarter	T + P
Bio19	Precipitation of Coldest Quarter	T + P

The subset of 6 variables selected based on the literature is formatted in bold.

#### Restricted set of six bioclimatic variables

Six variables were chosen among the 19 available bioclimatic variables using information about the insect biology. WCR is a soil inhabitant for most of its life cycle. Temperature and soil moisture are thus considered the most important abiotic parameters that could affect WCR establishment. Four parameters related to temperature and two parameters related to rainfall were assumed to be relevant for mapping the distribution of WCR (Table 1).

WCR metabolic activity and the development rate depend upon temperature. Degree days can be used to predict the life stages of this insect as shown by [40], [41], [42]. The Annual Mean Temperature was selected among the 19 available bioclimatic variables because this variable is related to the annual sum of degree days available for development. The « Minimum Temperature of the Coldest Month » and the « Mean Temperature of the Coldest Quarter » parameters were selected due to their possible effect on the mortality of eggs following exposure to low temperatures during a single month or on a longer period [43]. The Annual Temperature Range was also included because high death rates were found in the first and second larval instars [44] and temperature variation is known to be an important factor explaining high mortality of young larvae [45].

Ellsbury and Lee [46] suggested that wetness and temperature may both influence overwintering survival of WCR. Lack of winter precipitation can lead to a high mortality rate of eggs close to the soil surface [47]. This aspect was taken into account by selecting the variable: Precipitation of the Driest Quarter. Finally, the Precipitation of the Wettest Month was selected in order to take into account the influence of wetness on embryonic development after diapause termination [48] and the lack of oxygen in water-saturated soils.

#### Linear combinations of bioclimatic variables

Principal component analysis (PCA) was used to reduce the 19 bioclimatic variables (Table 1) into a smaller number of newly derived variables corresponding to independent linear combinations of the original variables. The basic principle of PCA is that, if there are some associations between the original variables, their first few linear combinations are able to explain most of the variation present in all the original variables [49]. The patterns in the original data can then be summarized into a much smaller linear combination of the variables than the full data set.

Principal components were extracted so that the first explained the maximum amount of variation in the 19 variables, the second the maximum amount of that unexplained by the first, etc. Following [49] (p. 443–454) the linear combinations, called principal components, were extracted by a spectral decomposition of the correlation matrix of the variables. The relationship between the individual bioclimatic variables and the extracted components was expressed by a Varimax rotated component matrix with Kaiser’s normalization, with components scaled between 0–1. The closer each component was to unity and further from zero, the greater contribution that variable made to that component. The number of components retained for further analyses was determined by the eigenvalue equals one rule [50] and scree diagram [49] (p. 452–453). Calculations were done in SPSS® v. 18.

### Model calibration

Nine models were used to predict the presence and absence of WCR in North America and Europe (Table 2). BIOCLIM is based on environmental envelope techniques [51], [52]. It first characterizes the environmental conditions of the actual distribution of the species and then identifies additional sites that fall within the already defined environmental hyperspace [53]. The Envelope Score algorithm (ES) is equivalent to the inclusive “OR” implementation of BIOCLIM described in [54]. DOMAIN assigns a value of habitat suitability to each potential site based on its proximity in the environmental space to the nearest occurrence location [55]. Environmental Distance (ED) is a generic algorithm based on environmental dissimilarity metrics. Climate Space Model (CSM) is based on a principle components analysis (PCA) technique where the optimum number of principal components is determined using the Broken-Stick cutoff method [56], [57]. The Genetic Algorithm for Rule-set Production (GARP) uses a genetic algorithm to select a set of mathematical rules defining the species ecological niche [58], [59]. Two versions of GARP were implemented in this study, the desktop version of GARP (DKGARP) and a new OpenModeller version (OMGARP) with an updated algorithm. MAXENT is a machine-learning method that estimates species distributions by finding the probability distribution of maximum entropy (i.e., that is most spread out, or closest to uniform) with constraints on the expected values of the environmental predictors [60], [61], [62]. Support Vector Machines (SVMs) methods [63] identifies an environmental envelope or hyperspace containing the data points, in which the envelope is optimized with respect to the number of points in the envelope and to the number of outliers [64].

**Table 2:** Nine models for predicting distribution of the western corn rootworm.

Name	Class of method	Data	Software
BIOCLIM	Envelope model	P	DIVA-GIS v5.2
Envelope Score (ES)	Envelope model	P	openModeller v1.0.9
DOMAIN	Multivariate distance	P	DIVA-GIS v5.2
Environmental Distance (ED)	Multivariate distance	P	openModeller v1.0.9
Climate Space Model (CSM)	Principal components analysis	P	openModeller v1.0.9
DKGARP	Genetic Algorithm for Rule Set Production, desktop version, with the best subset procedure	ppa	openModeller v1.0.9
OMGARP	Genetic Algorithm for Rule Set Production, openModeller version, with the best subset procedure	ppa	openModeller v1.0.9
MAXENT	Maximum Entropy	ppa	Maxent v3.3.1
Support Vector Machine (SVM)	Support Vector Machine	ppa	openModeller v1.0.9

Data needed for model calibration are presence data (p) or both presence and pseudo-absence data (ppa).

**Figure 3:**
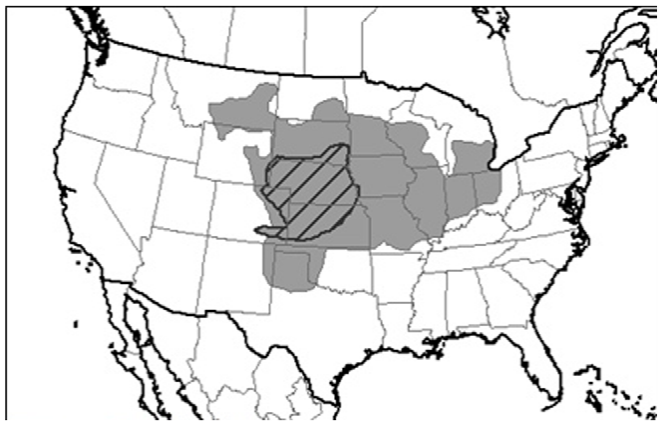
Geographical area of the training datasets. The hatched area represents the WCR distribution before 1955 while the grey area represents the WCR distribution before 1980.

BIOCLIM, ES, DOMAIN, ED and CSM only require presence sites for model calibration, whereas the two versions of GARP, MAXENT and SVM require both presence and pseudo-absence sites randomly sampled from the background. Twenty six training datasets were generated for model calibration as follows. Data were randomly selected from American presence data located in either i) the area where the species was recorded as present (the presence area) in USA before 1955 (Kansas, Colorado, Nebraska South Dakota), or ii) the presence area in USA before 1980 (the same states and Montana, Wyoming, North Dakota, New Mexico, Oklahoma, Texas, Minnesota, Wisconsin, Iowa, Missouri, Illinois, Michigan, Indiana, Ohio) (Figure 3).

Several sizes of training datasets were considered; 10, 20, and 51 (all) presence sites for the training datasets defined from the 1955 presence area, and 10, 20, 50, 100, 150, 300, and 428 (all) presence sites for training datasets defined from the 1980 presence area. Three replicates were generated for each training area and each sample size. Only the two training datasets including all presence sites (51 for the 1955 presence area and 428 for the 1980 presence area) were not replicated. This procedure allowed us to generate a range of training datasets with contrasting sizes corresponding to two different periods of the WCR invasion.

Each model was fitted to each training dataset using the three sets of bioclimatic variables in turn. BIOCLIM and DOMAIN were fitted using the DIVA-GIS software version 5.2 [65] (available at http://www.diva-gis.org), OMGARP, DKGARP, E S, ED, CSM and SVM were fitted using the OpenModeller software version 1.0.9 with default settings [66] (available at http://openmodeller.sourceforge.net) and MAXENT was fitted using the MAXENT software version 3.3.1 with default settings (available at http://www.cs.princeton.edu/~schapire/MAXENT/). When required, pseudo-absence data were generated using the software from the background (USA and Europe). The nine considered models are freely available, can be easily downloaded, and can be easily fitted to presence records using calibration algorithms from the website quoted above.

### Assessing model performance

Model performance was assessed with a test dataset that included the North American presence and absence sites and the European presence sites. The 428 North American presence sites used for model calibration were excluded from the test dataset in order to provide independent data for model assessment. The test dataset included 770 presence sites (240 in Europe) and 977 absence sites (all in North America).

The Receiver Operating Characteristic (ROC) methodology (e.g. [67], [68]) was used to evaluate the ability of each fitted model to discriminate between presence and absence sites. Two criteria were computed for each fitted model using the test dataset; likelihood ratio and the area under the ROC curve.

D is a binary variable equal to 1 for presence and to zero for absence. The test dataset was divided in two groups, one group including the presence sites and one group including the absence sites. Model outputs were computed for each site in both groups, and each model output value (O) was compared to a decision threshold (Th). The results were used to calculate the true positive proportion, defined as TPP (number of plots with O >Th in the group of sites with D = 1 divided by the total number of sites in this group) and the true negative proportion, defined as TNP (number of sites with O ≤ Th in the group of sites with D = 0 divided by the total number of sites in this group). TPP is referred to as Sensitivity. TNP is referred to as Specificity.

Sensitivity and specificity values have several practical uses. They can be directly used to study the accuracy of different indicators in relation to the decision threshold values. They can also be used to estimate other criteria such as the likelihood ratio ([69], [70]), defined as the ratio of Sensitivity to (1 - Specificity). This ratio can be used to compare the probability of correctly predicting a presence with the probability of incorrectly predicting a presence. The ratio should thus be as high a possible. A ratio close to one indicates that the two probabilities are similar and that the model is not very useful. Note that the likelihood ratio is dependent on the decision threshold-as well as the model. In pest risk assessment, decision makers are often primarily interested in high sensitivity values, in this study the likelihood ratio was therefore computed for a threshold Th leading to a sensitivity value equal to 0.95.

Sensitivity, specificity, and likelihood ratio depend on the decision threshold Th. These criteria provide useful information on the model accuracy in relation to the decision threshold chosen by the model user. Since different model users may consider different decision thresholds and since these thresholds are not necessarily known in advance, it is also useful to assess model accuracy for all possible decision thresholds. The ROC curve of a model is a graphical plot of Sensitivity against (1-Specificity), the values of TPP and TNP being calculated by varying the decision threshold Th over the whole range of values taken by the model output O. A summary of the overall accuracy of a model is the area under the ROC curve (AUC), that has an expected value of 0.5 for a non-informative model (i.e., a model no better than random classification) and of 1 for a perfect model (59). AUC has an important probabilistic interpretation. It is equal to the probability that the model outputs for randomly selected pairs of positive and negative events (here, D = 1 and D = 0) will be correctly ordered. In this study, a non-parametric estimate of the AUC value was calculated using the test dataset for all models and all training datasets.

The fitted models were ranked according to their AUC and likelihood ratio values. The effects of the size of the presence area (the presence area in 1980 compared to the presence area in 1955), the training dataset size (number of presence sites), and the set of input variables (19 bioclimatic variables, six bioclimatic variables, or the first three principal components) on model rank were tested using a non-parametric Wilcoxon statistical test. All computations were performed with R v.2.10 (cran.r-project.org).

## Results

### Linear combinations of bioclimatic variables

The first three linear combinations of bioclimatic variables explained 83.5% of the variance of the bioclimatic data. Removing the highly correlated bioclimatic variables and replacing them with the three uncorrelated linear combinations thus reduced the total variance explained by the bioclimatic variables by only 16.5%. The first linear component was attributed mainly to temperature, the second to precipitation during the wet or warm periods, and the third to precipitation during drought (Table 3).

**Table 3:** Rotated component matrix of Principal Component Analysis.

Bioclimatic variables	Components		
	1	2	3
Bio01 Annual Mean Temperature	0.967	0.212	−0.042
Bio02 Mean Diurnal Range (Mean of monthly (max temp - min temp))	0.403	−0.214	−0.379
Bio03 Isothermality (BIO2/BIO7) (* 100)	0.805	0.335	0.183
Bio04 Temperature Seasonality (standard deviation *100)	−0.843	−0.252	−0.267
Bio05 Max Temperature of Warmest Month	0.856	0.074	−0.287
Bio06 Min Temperature of Coldest Month	0.952	0.242	0.112
Bio07 Temperature Annual Range (BIO5-BIO6)	−0.773	−0.298	−0.365
Bio08 Mean Temperature of Wettest Quarter	0.764	0.328	−0.287
Bio09 Mean Temperature of Driest Quarter	0.958	0.11	0.095
Bio10 Mean Temperature of Warmest Quarter	0.892	0.148	−0.21
Bio11 Mean Temperature of Coldest Quarter	0.967	0.231	0.054
Bio12 Annual Precipitation	0.3	0.798	0.502
Bio13 Precipitation of Wettest Month	0.342	0.896	0.166
Bio14 Precipitation of Driest Month	0.095	0.382	0.82
Bio15 Precipitation Seasonality (Coefficient of Variation)	0.285	0.068	−0.745
Bio16 Precipitation of Wettest Quarter	0.332	0.894	0.205
Bio17 Precipitation of Driest Quarter	0.106	0.403	0.825
Bio18 Precipitation of Warmest Quarter	0.119	0.86	0.234
Bio19 Precipitation of Coldest Quarter	0.237	0.438	0.671

Varimax rotation method with Kaiser normalization. The components are scaled between 0–1; the closer the values to one, the more variance they explain. Values between 0.7–0.79, 0.8–0.89.

### AUC

Table 4 shows the significance of the effects of the presence area (1980 vs. 1955), the size of the training dataset, and the set of bioclimatic variables on AUC values.

**Table 4:** Significance of effect of training area, size of training dataset, and set of bioclimatic variables on AUC values.

Model	Area 1980/1955	Input variables						Size		
		6/19var		PCA/19var		PCA/6var		20/10		Big/Small
		1955	1980	1955	1980	1955	1980	1955	1980	
BIOCLIM	***	*	***	**	***	**	**	NS	NS	***
CSM	***	NS	NS	NS	NS	NS	NS	NS	NS	NS
DKGARP	.	***	***	***	***	**	***	NS	NS	***
DOMAIN	***	NS	NS	NS	***	NS	***	NS	NS	NS
ED	***	NS	NS	NS	***	NS	***	NS	NS	NS
ES	NS	NS	NS	NS	NS	NS	NS	NS	NS	***
MAXENT	***	NS	NS	***	***	***	***	NS	NS	NS
OMGARP	.	***	***	***	***	NS	.	NS	NS	NS
SVM	***	NS	NS	NS	*	.	*	NS	NS	NS

Area 1980 vs. 1955, 6 variables vs. 19 variables, first three principal components (PCA) vs. 19 variables, PCA vs. 6 variables, training dataset size  =  20 vs. 10, big training dataset (more than 50 presence points) vs. small (less than 50).

***p<0.001 |

**p<0.01 |

*p<0.05 |. p<0.1 | NS not significant.

**Figure 4:**
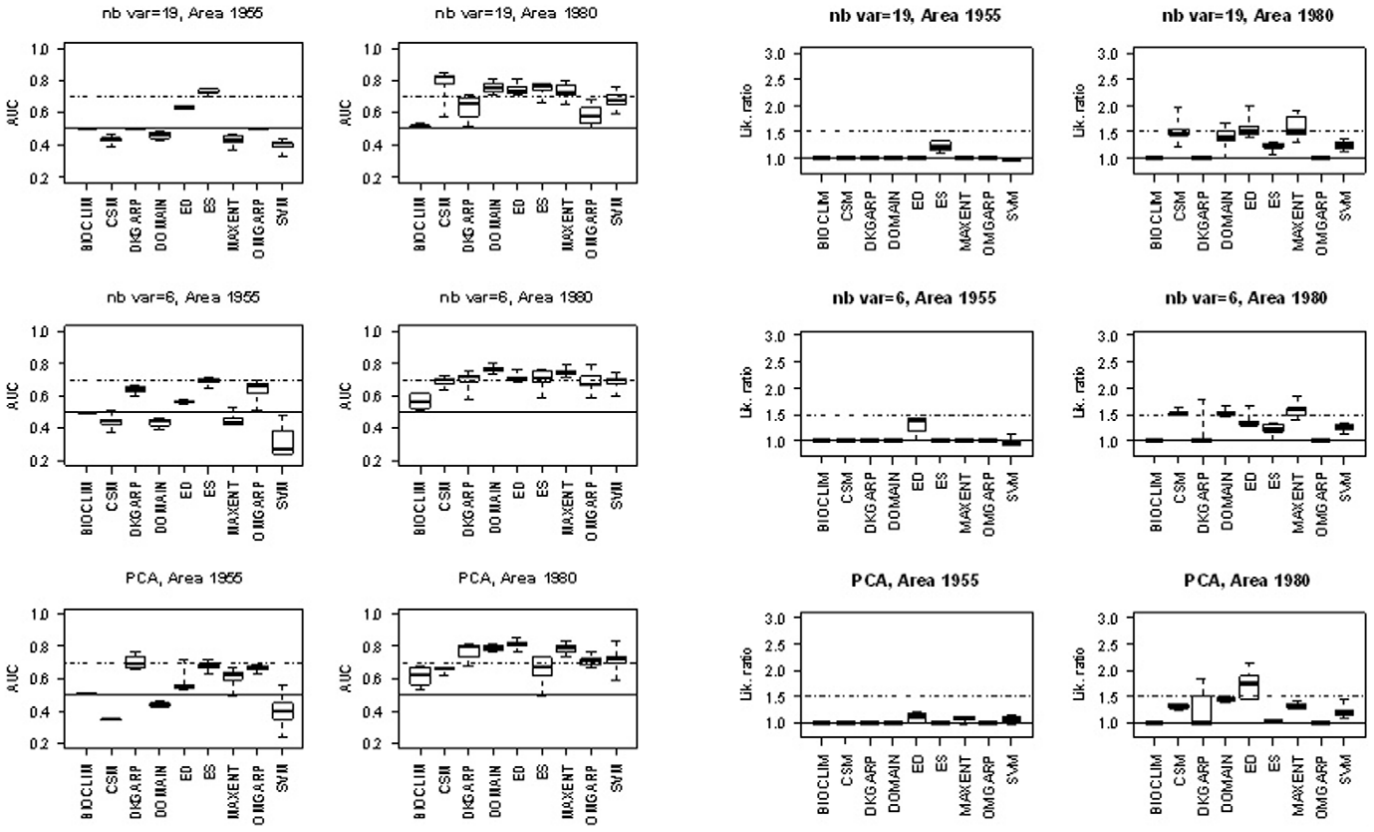
Box plots of AUC values and likelihood ratios (sensitivity = 0.95) computed for the nine models with 19 variables, 6 variables, or three principal components (PCA) for training datasets generated from two areas (1955 and 1980). Continuous and dashed lines correspond to AUC = 0.5 or ratio = 1 and AUC = 0.7or ratio = 1.5 respectively.

**Figure 5:**
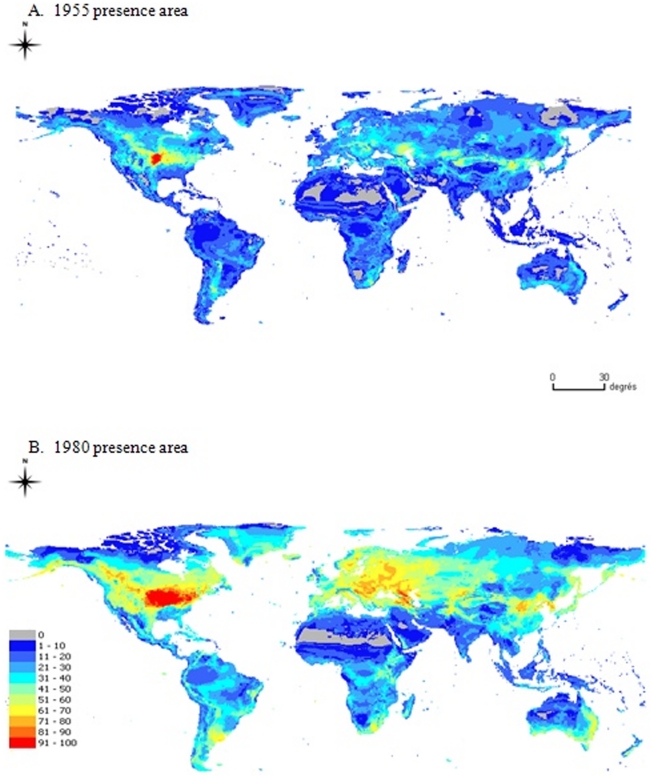
Outputs of model ES obtained with 20 presence data located within the 1955 presence area (A) and with 50 presence data located within the 1980 presence area (B). The model was fitted using 19 input variables in both cases.

For all models except DKGARP, OMGARP and ES, AUC values were significantly higher with the 1980 presence area than with the 1955 presence area (*p* = 0.05). The strong influence of the presence area on AUC is confirmed by the box plots displayed in Figure 4; AUC were more frequently higher than 0.7 with the 1980 presence area than with the 1955 presence area. However, even with the 1980 presence area, the AUC was higher than 0.8 in a limited number of cases. The influence of the presence area on model outputs is illustrated for model ES in Figure 5; the levels of risk predicted by ES were highly dependent on the presence area used to calibrate the model.

The selection of the bioclimatic input variables also had a significant effect on model AUC (Table 4). The effects of the input variables on AUC are visible in Figure 4, but the box plots show that the effect of this factor was smaller than the effect of the presence area. The use of 6 bioclimatic variables instead of 19 significantly increased the AUC of BIOCLIM, DKGARP and OMGARP. The use of the first three principal components significantly increased the AUC when the model was fitted to training datasets generated from the 1980 presence area. The only exceptions were CSM and ES; the AUC values of these two models were not influenced by the type of input variables (Table 4).

The effect of the size of the training dataset was not significant for the 1955 presence area; the AUC values of models fitted to 10 presence sites and to 20 presence sites were similar (Table 4). For the 1980 presence area, training dataset size was significant for three models; BIOCLIM, DKGARP, and ES (Table 4). The AUC values of these models were increased by using a training dataset including more than 50 presence sites.

Table 5 shows that the results of the comparison of AUC values with two thresholds, 0.5 and 0.7. With the 1955 presence area, values obtained for CSM, DOMAIN and SVM were never significantly higher than 0.5 and only ES with the 19 bioclimatic variables showed an AUC value significantly higher than 0.7 with this restricted area (Table 5, Figures 4–5).

**Table 5:** Significance of the model performance.

Model	Input variables	AUC = 0.5		AUC = 0.7		LikR = 1		LikR = 1.5	
		1955	1980	1955	1980	1955	1980	1955	1980
BIOCLIM	6	.	***	NS	NS	NS	NS	NS	NS
	19	NS	***	NS	NS	NS	NS	NS	NS
	3 PCA	*	***	NS	NS	NS	NS	NS	NS
CSM	6	NS	***	NS	NS	NS	***	NS	**
	19	NS	***	NS	***	NS	***	NS	NS
	3 PCA	NS	***	NS	NS	NS	***	NS	NS
DKGARP	6	**	***	NS	.	NS	.	NS	NS
	19	NS	***	NS	NS	NS	NS	NS	NS
	3 PCA	**	***	NS	***	NS	**	NS	NS
DOMAIN	6	NS	***	NS	***	NS	***	NS	*
	19	NS	***	NS	***	NS	***	NS	NS
	3 PCA	NS	***	NS	***	NS	***	NS	NS
ED	6	**	***	NS	*	*	***	NS	NS
	19	**	***	NS	***	NS	***	NS	NS
	3 PCA	**	***	NS	***	.	***	NS	**
ES	6	**	***	NS	NS	NS	***	NS	NS
	19	**	***	*	***	**	***	NS	NS
	3 PCA	**	***	NS	NS	NS	***	NS	NS
MAXENT	6	NS	***	NS	***	NS	***	NS	**
	19	NS	***	NS	***	NS	***	NS	.
	3 PCA	*	***	NS	***	*	***	NS	NS
OMGARP	6	**	***	NS	NS	NS	NS	NS	NS
	19	NS	***	NS	NS	NS	NS	NS	NS
	3 PCA	**	***	NS	.	NS	NS	NS	NS
SVM	6	NS	***	NS	NS	NS	***	NS	NS
	19	NS	***	NS	NS	NS	***	NS	NS
	3 PCA	NS	***	NS	.	.	***	NS	NS

Tests “AUC<0.5 vs. AUC>0.5”, “AUC<0.7 vs. AUC>0.7”, “Likelihood ratio<1 vs. Likelihood ratio>1”, and “Likelihood ratio<1.5 vs. Likelihood ratio>1.5”.

***p<0.001 |

**p<0.01 |

*p<0.05 |. p<0.1 | NS not significant.

With the 1980 presence area, AUC was significantly higher than 0.5 for all models and all types of input variables. With this area, AUC was significantly higher than 0.7 for several models; DOMAIN, ED, and MAXENT with all types of input variables, CSM and ES with 19 bioclimatic variables, and DKGARP with the first three principal components (Table 5). With the 1980 presence area, the AUC values of BIOCLIM, OMGARP, and SVM were never significantly higher than 0.7 (*p* = 0.05) (Table 5).

### Likelihood ratios

Table 6 shows the significance of the effects of presence area, size of training dataset, and set of bioclimatic variables on likelihood ratios.

**Table 6:** Significance of effect of training area, size of training dataset, and set of bioclimatic variables on likelihood ratio values (sensitivity = 0.95).

Model	Area 1980/1955	Input variables						Size		
		6/19var		PCA/19var		PCA/6var		20/10		Big/Small
		1955	1980	1955	1980	1955	1980	1955	1980	
BIOCLIM	NS	NS	NS	NS	NS	NS	NS	NS	NS	NS
CSM	***	NS	.	NS	NS	NS	NS	NS	NS	NS
DKGARP	.	NS	*	NS	***	NS	*	NS	NS	*
DOMAIN	***	NS	*	NS	NS	NS	NS	NS	NS	NS
ED	***	**	NS	*	.	NS	***	NS	NS	NS
ES	NS	NS	NS	NS	NS	NS	NS	NS	.	**
MAXENT	***	NS	NS	*	NS	*	NS	NS	NS	NS
OMGARP	NS	NS	NS	NS	NS	NS	NS	NS	NS	NS
SVM	***	NS	NS	*	NS	*	NS	NS	NS	NS

Area 1980 vs. 1955, 6 variables vs. 19 variables, first three principal components (PCA) vs. 19 variables, PCA vs. 6 variables, training dataset size  =  20 vs. 10, big training dataset (more than 50 presence points) vs. small (less than 50).

***p<0.001 |

**p<0.01 |

*p<0.05 |. p<0.1 | NS not significant.

For all models except BIOCLIM, DKGARP, OMGARP and ES, likelihood ratios were significantly higher with the 1980 WCR presence area than with the 1955 area (*p* = 0.05). The influence of the presence area on likelihood ratios is confirmed by the box plots displayed in Figure 4. Ratios were more frequently higher than the thresholds of 1 and 1.5 with the 1980 presence area than with the 1955 presence area. However, even with the 1980 presence area, likelihood ratios were higher than two in a limited number of cases.

The type of the input variables had no significant effect on the likelihood ratios of BIOCLIM, CSM, ES, and OMGARP (Table 6). The use of 6 bioclimatic variables instead of 19 significantly increased the likelihood ratio for DKGARP (with the 1980 area), DOMAIN (with the 1980 area), and ED (with the 1955 presence area). The use of the first three principal components significantly increased the likelihood ratio of DKGARP (with the 1980 presence area only), ED, MAXENT and SVM (with the 1955 presence area only).

For the 1955 presence area, the size of the training dataset had no significant influence on the likelihood ratio. For the 1980 presence area, the size of the training dataset had a significant effect only for DKGARP and ES; the likelihood ratios of these models were significantly higher when more than 50 presence sites were used for model calibration.

Table 5 shows the results of the comparison of likelihood ratios with two thresholds, 1 and 1.5. For the 1955 presence area, only ratios obtained for ED (with 6 variables), ES (with 19 variables), and MAXENT (with the first three principal components) were significantly higher than 1 and ratios were never significantly higher than 1.5 with this restricted area. For the 1980 presence area, all models showed ratios significantly higher than 1 with two exceptions, BIOCLIM and OMGARP. For this large presence area, the likelihood ratio was significantly higher than 1.5 in a few cases: CSM with 6 input variables, DOMAIN with 6 input variables, ED with the first three principal components and MAXENT with 6 input variables.

## Discussion

According to Peterson [8], ecological niche models assess only one step of a species invasion; the ecological appropriateness of new landscapes. Predicting the current or future distributions of species is principally conducted using bioclimatic models. These models assume that climate ultimately restricts species distributions. This assumption is made because in most situations climate is the only factor for which data are readily available as the input variables; however, in reality, the distribution is also under the influence of other environmental and ecological components (e.g., altitude, host presence, competition, predation).

Our results showed that the performance of species distribution models was highly variable and depended on the extent of the species presence area, the size of the training dataset, and the type and number of bioclimatic input variables. In almost all the conditions tested, the AUC was not significantly higher than 0.7 and the likelihood ratio was not significantly higher than 1.5 when training datasets were defined from the 1955 presence area. This result shows that the models tested were not very useful for predicting presence/absence of an invasive species, *Diabrotica virgifera virgifera* when it was at an early stage of an invasion. Model performance was much better when models were run with training datasets generated from a larger presence area corresponding to the WCR presence in USA in 1980. The models tested seem to be better able to predict invasive species establishment when the species has been recorded in a relatively large area and thus is more likely to have reached its climatic limits. However, in this case, results show that model performance depends on model type, the number of bioclimatic input variables, and the size of the training dataset.

Among the nine models considered in this study, BIOCLIM, OMGARP, and SVM showed poor performance for the two presence areas (1955 and 1980) and the three sets of bioclimatic input variables (19, 6 and the three principal components). The AUC of these models was never significantly higher than 0.7 and their likelihood ratios were never significantly higher than 1.5 (p = 0.05). In addition, the likelihood ratios of BIOCLIM and OMGARP were never significantly higher than 1, *i.e.* the probability of correct presence prediction was thus not higher than the probability of incorrect presence prediction when these models were used to predict presence of WCR in North America and Europe.

With the other six models (CSM, DKGARP, DOMAIN, ED, ES, MAXENT), the AUC and the likelihood ratio were higher than 0.7 and 1 in at least some of the conditions tested. For three of these models (DOMAIN, ED, and MAXENT), the AUC was significantly higher than 0.7 for all the three sets of bioclimatic input variables when training datasets were generated from the 1980 presence area. In addition, the likelihood ratio of these three models was significantly higher than 1.5 in some cases. The performance of these models seems quite robust with the 1980 presence area. The model ES also showed good performance with 19 bioclimatic variables and ES is the only model giving an AUC significantly higher than 0.7 when training datasets were generated from the 1955 presence area. Overall, DOMAIN, ED, MAXENT, and ES (with 19 bioclimatic variables) were the most accurate models. The good performance of MAXENT is likely due to its regularization procedure that counteracts a tendency to over-fit models when using few species occurrences [21], [60]. Its amount of regularization varies flexibly with sample size to ensure consistent performance [22]. According to Giovanelli et al. [28], GARP algorithms are penalized by their intrinsic measure of predictive accuracy that tends to fail to discriminate the best among a number of alternative models. Elith and Graham [71] showed that GARP tended to overpredict suitability of invasive species. The BIOCLIM model was found to perform poorly in several past studies e.g., [21], [22]. This model performed also poorly in our comparative study, but the version of BIOCLIM including the “OR” option (i.e., the ES algorithm) performed well with small samples when a large number of input variables was considered.

Although other studies found that several distribution models could show AUC values higher than 0.9 (e.g., [21], [28]), such results were not obtained in our study; the AUC values never reached 0.9 and the likelihood ratio was very rarely higher than 2 among all the conditions tested. All models thus led to a substantial rate of mis-classification and pest risk assessors need to keep this in mind when using these models to predict species distributions.

One of the reasons of mis-classification may be the use of either too few or too many bioclimatic variables [29], [30], [33], [34]. The inclusion of too few variables can over estimate species distributions by excluding from the model those variables that restrict the species; in turn, inclusion of too many variables may lead to mis-representations due to over-fitting because the progressive addition of variables can result in progressively smaller potential distributions. The inclusion of unnecessary variables may also place unrealistic constraints on identifying climatically suitable habitat, and thus may result in areas being classified as climatically unsuitable when, in fact, they are appropriate.

The significance of the number of bioclimatic variables found in this paper demonstrates that the number of model input variables is an important consideration. With the 1980 presence area, the use of the three linear combinations of the 19 variables significantly improved model-based classification for seven out of nine models. This is because the three principal axes merged together information from the original 19 bioclimatic variables with little lost from the variability of the original variables. The increase in precision can thus be attributed to the removal of redundant parameters, which prevents over-fitting. Compared to principal component analysis, the benefit of using six bioclimatic selected from the literature was smaller. This may be due to the difficulty of identifying relevant variables using information about the insect biology and, also, to the relatively small number of bioclimatic variables available in our climatic dataset.

Several of the models tested in this study used ‘pseudo-absence’ data for calibration. Performance of SDMs was found to be sensitive to the type of pseudo-absence data used for calibration [32]. When pseudo-absence data are used in SDMs, the area from which the pseudo-absence points are derived influence the outcome of models. As only one type of pseudo-absence data was considered in our comparative study, it will be interesting to see how our conclusions are changed when pseudo-absence data are generated with alternative techniques proposed by [32].

The methodological framework and the datasets presented in this paper could be used to assess other models or other model settings in the future, for example models based on machine learning techniques like regression tree and random forest, and more mechanistic models such as NAPPFAST. It would be also informative to implement the same methodology with other invasive species.
